# Nanoparticle Vaccines Based on the Truncated VZV gE Elicit a Robust Immune Response in Mice

**DOI:** 10.3390/vaccines14010069

**Published:** 2026-01-07

**Authors:** Tianxin Shi, Hai Li, Jiehui Wu, Hongqiao Hu, Jie Jiang, Ruichen Wang, Ziyi Li, Qianqian Cui, Shihong Fu, Kai Nie, Fan Li, Qikai Yin, Huanyu Wang, Songtao Xu

**Affiliations:** 1 National Key Laboratory of Intelligent Tracking and Forecasting for Infections Disease, National Institute for Viral Disease Control and Prevention, Beijing 102206, China; shitianxin2022@163.com (T.S.); lihai@ivdc.chinacdc.cn (H.L.); wujiehui5439@163.com (J.W.); huhq049@163.com (H.H.); jiejiang0317@163.com (J.J.); wangrc96@163.com (R.W.); liziyi_99@163.com (Z.L.); cuiqq@ivdc.chinacdc.cn (Q.C.); fush@ivdc.chinacdc.cn (S.F.); niekai@ivdc.chinacdc.cn (K.N.); lifan@ivdc.chinacdc.cn (F.L.); yinqk@ivdc.chinacdc.cn (Q.Y.); wanghy@ivdc.chinacdc.cn (H.W.); 2 China Medical University, Shenyang 110122, China

**Keywords:** varicella zoster virus, glycoprotein E, nanoparticle, ferritin

## Abstract

**Background:** Herpes zoster (HZ), caused by the reactivation of varicella-zoster virus (VZV), primarily affects elderly populations worldwide. Although current recombinant HZ vaccines show strong immunogenicity, their high cost and potential side effects may limit their widespread use. Therefore, developing a cost-effective HZ vaccine with improved safety profiles would have significant clinical and public health implications. **Methods:** Building upon our previously optimized truncated gE (tgE350) from VZV, we developed the tgE350 + Fe nanoparticle vaccine using SpyTag/SpyCatcher covalent conjugation. The tgE350 protein (with a SpyTag tag) and the Fe protein (with a SpyCatcher tag) were expressed in HEK293F and *E. coli* BL21, respectively, enabling spontaneous nanoparticle assembly. Protein expression and nanoparticle formation were confirmed through SDS-PAGE and negative-stain electron microscopy. BALB/c mice were inoculated with either tgE350 + Fe or tgE350 combined with Al and CpG adjuvants. Immune responses were evaluated using ELISpot and flow cytometry for cellular immunity, along with ELISA, VZV microneutralization, and fluorescent antibody membrane antigen (FAMA) assays for antibody titers. Histopathological examination of major organs ensured vaccine safety. **Results:** Compared with the truncated vaccine tgE350, the nanoparticle vaccine tgE350 + Fe significantly enhanced VZV neutralizing antibodies and specific antibody responses in mice without causing significant changes in lymphocyte populations (no difference from the control group). Moreover, the tgE350 + Fe group had significantly more lymphocytes secreting IFN-γ, IL-2, and IL-4 than the tgE350 group. No apparent pathological damage was observed in the heart, liver, spleen, or lungs of mice in any experimental group. **Conclusions**: This experiment successfully developed the HZ nanoparticle vaccine tgE350 + Fe. It enhanced VZV-specific neutralizing antibodies, generated better cellular and humoral immune responses, and demonstrated good safety.

## 1. Introduction

Varicella-zoster virus (VZV), a member of the human herpesvirus family, has typical latent and then reactivated properties [1]. At the time of initial infection, the virus spreads through the respiratory tract to cause varicella (chickenpox) [2], and then migrates along the sensory nerves to the dorsal root ganglia to establish a lifelong latent infection. As host immunity wanes, the latent virus can reactivate and disseminate along nerve axons, precipitating a vesicular rash with characteristic dermatomal distribution and severe neuralgia, known as herpes zoster (HZ) [3]. This condition has been shown to result in a substantial decline in patients’ quality of life. Epidemiological data demonstrate that the global prevalence of varicella-zoster virus (VZV) infection in adults is more than 90% [1], and the disease burden continues to increase. In 2019, there were 83.96 million new cases of VZV-associated diseases globally, representing an increase of 17.85% from a decade ago [4]. This results in billions of dollars in direct healthcare costs and lost productivity each year. It is noteworthy that the pathogenicity pattern of the virus displays a bimodal distribution, with children under 5 years of age being susceptible to chickenpox, and the elderly population over 50 years of age constituting the primary affected group of HZ due to age-related immune senescence [5]. The expansion of the immunosuppressed population and the superposition of stressors during the ongoing pandemic of the novel severe acute respiratory syndrome (SARS-CoV-2) virus have further led to a surge in the number of reported cases of HZ globally [6], underscoring the need for urgent measures to ensure its public health prevention and control.

Among the multiple proteins encoded by varicella-zoster virus (VZV), glycoprotein gE (ORF68) is a key target for vaccine development due to its multiple biological functions [7]. As the most abundant glycoprotein in the viral envelope, gE not only synergizes with gB to mediate cell membrane fusion, but also facilitates direct cell-to-cell spread of the virus by binding to gI to form a complex and participates in intracellular transport of gH/gL [8,9,10]. Its strong immunogenicity makes it an ideal antigen for subunit vaccine design. For instance, the Shingrix vaccine, which combines gE with the AS01_B_ adjuvant, has demonstrated more than 90% protective efficacy in elderly populations by activating CD4^+^ T cells and high-affinity neutralizing antibodies, thus marking a significant milestone in the prevention and control of herpes zoster (HZ). Moreover, studies utilizing animal models have shown that gE deletion significantly inhibits viral replication in SCID mice, further validating its importance as a therapeutic target [11,12]. However, existing vaccine platforms still face significant challenges. Zostavax, a live attenuated vaccine marketed in 2005, is able to activate T-cell immunity, but its efficacy declines sharply with age, with a protection rate of only 38% in people aged 70 years or older, a limitation that highlights the inadequacy of live attenuated vaccines for patient protection [7,13]. The Shingrix vaccine, approved in 2017, demonstrates high efficacy in the elderly. However, its widespread adoption is constrained by injection-site reactions attributable to the AS01_B_ adjuvant system, alongside relatively high production costs. Statistical studies have demonstrated that the utilization of the herpes zoster vaccine results in a 61.1% reduction in the burden of disease caused by herpes zoster, a 66.5% reduction in the incidence of postherpetic neuralgia, and a 51.3% reduction in the incidence of herpes zoster [14]. At present, herpes zoster vaccination is regarded as the most cost-effective solution.

Innovations in vaccine technology are closely linked to breakthroughs in adjuvants and nanotechnology. Aluminum adjuvants are widely used in vaccines such as diphtheria-tetanus-pertussis (DTP) and Hepatitis B due to their established safety profile [15]. The TLR9 agonist CpG ODN can activate Th1 immune responses and cytotoxic T cells, significantly enhancing the effectiveness of Hepatitis B and malaria vaccines, particularly in HIV-infected individuals [12,15]. In recent years, research on nanoparticle-based vaccine platforms has advanced significantly. Among these, nanoparticle vaccines constructed using self-assembling ferritin have become a priority in vaccinology. Ferritin naturally forms a hollow spherical structure composed of 24 subunits. Through genetic engineering, it can be adapted as an antigen display platform to present antigens such as the influenza HA protein or the HIV gp120 protein, thereby significantly enhancing immunogenicity [16,17]. Studies show that a ferritin nanoparticle-based influenza vaccine induces higher levels of neutralizing antibodies than conventional inactivated vaccines in mouse models. Its good biocompatibility and degradability provide a foundation for the design of next-generation vaccines. These advances offer new strategies for optimizing herpes zoster (HZ) vaccines: by leveraging antigen display technology based on self-assembling nanoparticles like ferritin, combined with the synergistic effect of potent adjuvants, it is possible to further enhance vaccine-induced humoral and cellular immune responses [18].

The currently marketed HZ recombinant protein vaccine has good immunogenicity but is limited by its high cost and side effects. Therefore, the development of a new HZ vaccine with few side effects and low cost is of great clinical value and public health significance. In this study, we constructed a VZV nanoparticle vaccine based on pre-screened VZV truncated gE antigen (tgE350), combined with ferritin nanoparticles, using SpyTag/SpyCatcher covalent linkage system, and immunized BALB/c mice in combination with Al and CpG adjuvants to compare the humoral and cellular immune responses induced by tgE350 and tgE350 + Fe. The mice were also analyzed for damage to various organs to ensure the safety of the vaccine. This study aims to provide new technical ideas for the design of next-generation HZ vaccines and to promote the development of better vaccine strategies.

## 2. Materials and Methods

### 2.1. Cells and Animals

Well-maintained human embryonic kidney 293F cells (HEK 293F) were selected and mixed with 90% fetal bovine serum and 10% dimethyl sulfoxide (DMSO), resulting in a cell concentration of 2.5 × 10^6^ cells/mL. The cell suspension was aliquoted into cryovials (1–2 mL each) and stored in liquid nitrogen for preservation in our laboratory (Arbovirus Laboratory, Chinese Center for Disease Control and Prevention). Specific pathogen-free female BALB/c mice, aged 6–8 weeks, were purchased from SPF biotechnology Co., Ltd. (Beijing, China).

### 2.2. Recombinant Plasmid Extraction, Expression and Purification

Ferritin (Fe) was expressed using *Escherichia coli* (*E. coli*). A 6× His tag was incorporated at the C-terminus of DNA sequence encoding the human ferritin (GenBank: NC_000011.10). The SpyCatcher peptide sequence was fused to the N-terminus of human ferritin. The resulting fragment, containing a termination codon (TAATAG), was cloned into the pET-28(+) vector via EcoRI and NotI restriction sites. The plasmid was transformed into the BL21 strain, and the transformed cells were then cultured in LB medium without antibiotics at 37 °C with shaking at 180 rpm. A total of 100 μL of the transformed culture was spread on LB plates containing kanamycin (kana+) and incubated overnight at 37 °C. Well-isolated monoclonal colonies were selected and transferred to LB medium containing kanamycin, then cultured at 37 °C and 180 rpm until the optical density (OD) reached approximately 0.6. At this point, isopropyl β-D-1-thiogalactopyranoside (IPTG) was added, and the culture was then incubated with shaking at 180 rpm. After 18 h of incubation, the culture was centrifuged, and the pellet was resuspended in buffer. Ultrasonication was then performed to release the protein, and the supernatant was retained following centrifugation. The Fe protein was purified using Ni-NTA affinity chromatography with a liquid chromatography system (Cytiva, AKTA Pure, Marlborough, MA, USA). The protein solution was concentrated using an ultrafiltration tube and exchanged with a conventional buffer. Finally, the protein concentration was determined using the BCA assay.

The pcDNA3.4-ST-tgE350 expression plasmid was constructed by engineering a truncated form (amino acids 1-350) of human alphaherpesvirus 3 gE glycoprotein (GenBank NC_001348.1), removing its intracellular domain, transmembrane region, and partial extracellular structures. The modified sequence was fused with an N-terminal SpyTag peptide and a C-terminal 6× His tag, codon-optimized, and cloned into the pcDNA3.4 vector using BsmBI and XbaI restriction sites to create the final expression construct. The *E. coli* DH5α strain harboring the plasmid was inoculated in LB medium containing ampicillin (Amp+) and cultured overnight at 37 °C with agitation at 180 rpm. After centrifugation, the pellet was collected. Plasmid extraction was performed using the QIAGEN Plasmid Maxi Kit (QIAGEN, Cat. #12163, Hilden, Germany). Following the requirements of the HEK293F cell transfection kit, the plasmid was introduced into HEK293F cells for expression. When cell viability decreased to approximately 80%, the cells were harvested, centrifuged, and the supernatant was collected. The tgE350 protein was purified using Ni-NTA affinity chromatography. The solution was concentrated with an ultrafiltration device and exchanged with a conventional buffer. Finally, protein concentration was quantified using the BCA assay.

### 2.3. Size-Exclusion Chromatography (SEC)

The ST-tgE350 protein was incubated with SC-Fe protein at a 1:1 molar ratio in PBS buffer on a shaker at 4 °C and 30 rpm for 16 h. Next, we performed size-exclusion chromatography to separate coupled nanoparticles from unbound proteins. The eluted protein from the first peak will represent the coupled nanoparticles. We will further concentrate the protein using ultrafiltration tubes and measure the concentration with the BCA assay. After purification by affinity chromatography and gel filtration, endotoxin levels are significantly reduced, effectively eliminating it as a confounding variable in the analysis.

### 2.4. Negative Staining Electron Microscopy (EM)

Fe and tgE350 + Fe nanoparticles proceeded to negative staining electron microscopy (EM) using an instrument from Shuimu BioSciences Corporation (Beijing, China). A 3 μL sample solution is deposited onto a copper mesh (mesh size of 230) with carbon-coated grid, which had been hydrophilically treated with a glow discharger. After incubating for 60 s, excess sample solution is blotted from the edge of the grid with filter paper (avoiding grid dryness). The grid is then washed twice with 3 μL of 2% uranyl acetate solution. Subsequently, it is stained with 3 μL of the same solution for 60 s, and excess stain is removed from the edge of the grid with filter paper. After being naturally dried, the grid is observed using a transmission electron microscope (ThermoFisher Tecnai Spirit, 120 kV, Waltham, MA, USA) equipped with an EMSIS Veleta camera (2K × 2K) (Münster, Germany).

### 2.5. Animal Vaccination

Specific pathogen-free (SPF) female BALB/c mice (6–8 weeks old, 16–18 g) were randomly divided into five groups (*n* = 6 per group) for immunization. Mice in the two experimental groups received intramuscular (i.m.) injections into both thigh muscles (50 μL per thigh, 100 μL total). The tgE350 group was immunized with a formulation containing 10 μg of tgE350 protein adjuvanted with 50 μg aluminum (Al) and 10 μg CpG ODN 2395. The tgE350 + Fe group received an equivalent protein dose of tgE350 + Fe nanoparticles with the same adjuvant. Three control groups received identical-volume (100 μL) i.m. injections under the same schedule. The Mock group was injected with PBS. The Fe group received a ferritin solution containing an amount of Fe equivalent to that in the tgE350 + Fe nanoparticles. The Al/CpG group received the adjuvant mixture alone (50 μg Al + 10 μg CpG).

All mice were immunized on days 0 and 21. Blood was collected from the mandibular area 14 days after the prime immunization and via the retro-orbital plexus 14 days after the boost. Serum was separated by centrifugation (2000 rpm, 40 min) and stored at −80 °C for ELISA. Spleens were harvested for single-cell suspension preparation to perform ELISpot and flow cytometry. Hearts, livers, spleens, and lungs were collected for histopathological sectioning and hematoxylin-eosin (H&E) staining.

### 2.6. ELISpot

For ELISpot, fresh spleens were obtained from mice and promptly homogenized to generate a single-cell suspension. We washed the pre-coated ELISpot plates with anti-mouse IFN-γ (Mabtech, cat. #3321-4HST-2, Nacka, Sweden), IL-4 (Mabtech, cat. #3311-4HPW-2, Sweden), and IL-2 (Mabtech, cat. #3441-4APW-2, Sweden) antibodies, incubated them with culture medium, and then added the protein stimulant gE (10 μg/mL), followed by the positive control reagent (PMA^+^ Ionomycin) and 1640 medium. Finally, we overlaid the plates with splenocytes (5 × 10^5^ cells/well). The plates were incubated at 37 °C with 5% CO_2_ for 48 h. After washing, the corresponding detection antibodies were added according to the manufacturer’s protocol. Following another wash, a diluted streptavidin–ALP solution was applied, and after a subsequent wash, the BCIP/NBT substrate solution was added. Finally, the plates were rinsed with deionized water, dried, and then counted using the Mabtech IRIS FluoroSpot/ELISpot reader.

### 2.7. Flow Cytometry

The prepared splenic single-cell suspension was adjusted to a concentration of 107 cells/mL with PBS. A volume of 100 μL of this suspension was transferred to a flow cytometry tube, to which 2 mL of PBS was added. The mixture was vortexed and centrifuged at 300× *g* for 5 min, after which the supernatant was discarded. Simultaneously, blank controls, individual tubes for each fluorescently labeled antibody and viability dye, and the necessary isotype control tubes were prepared. The FVS700 was dissolved in DMSO and then diluted to 1:1000 with PBS. A volume of 1 mL of the diluted staining solution was added to each sample tube, and the mixture was incubated at room temperature in the dark for 15 min. The cells were mixed with PBS containing 1% FBS and centrifuged, discarding the supernatant. They were then resuspended in 100 μL of PBS with 1% FBS, and 1 μL each of CD45 FITC (BioLegend, cat. #103108, San Diego, CA, USA), CD3 APC-Cy7 (BioLegend, cat. #100222, San Diego, CA, USA), CD4 PerCP-Cy5.5 (BioLegend, cat. # 100433, San Diego, CA, USA), CD8 APC (BioLegend, cat. # 162306, San Diego, CA, USA), CD44 PE-Cy7 (BioLegend, cat.#103030, San Diego, CA, USA), and CD62L BV421 (BioLegend, cat. # 104436, San Diego, CA, USA) was added. After mixing, the samples were incubated at room temperature in the dark for 20 min. Then, PBS containing 1% FBS was added, mixed, and centrifuged to discard the supernatant. Following this, 500 μL of PBS with 1% FBS was added and mixed. Data were acquired using the SpectroFloCLC 1.0 software on the Cytek NL-CLC3000 (Fremont, CA, USA) flow cytometer, and data analysis was performed using FlowJo V10.8 software.

### 2.8. Enzyme-Linked Immunosorbent Assay (ELISA)

gE protein diluted in PBS was added to the ELISA plate and incubated overnight at 4 °C. The following day, the plate was washed with PBST, and blocking solution was applied, followed by a 2 h incubation at 37 °C. After another wash with PBST, diluted serum was added (1:200 dilution for the first row of a 96-well plate, with subsequent wells diluted 4-fold) and incubated for 2 h at 37 °C. Following another wash with PBST, HRP-conjugated goat anti-mouse IgG (1:5000), IgG1 (1:2000), and IgG2a (1:2000) were added at 100 μL/well and incubated for 30 min at 37 °C. After washing with PBST, the ELISA termination solution was added. Finally, absorbance was measured at 450 nm using a microplate reader.

### 2.9. In Vitro Microneturalization Assay

Vazyme Biotech Co., Ltd. (Nanjing, China) was commissioned for a microneutralization assay. MRC-5 cells were seeded in 96-well plates and incubated overnight at 37 °C in a 5% CO_2_ environment. Serum samples were heat-inactivated at 56 °C for 30 min, initially diluted 30-fold, followed by three additional three-fold dilutions, yielding eight dilution levels, each tested in duplicate. After diluting, the virus was added to sample and control wells for one hour of neutralization at 37 °C. Then, 50 μL of the virus-serum mixture, positive controls, and back-titration samples were added to the cell monolayers. After a 2 h incubation, the medium was replaced, and cells were incubated for 48 h. The supernatant was removed, cells fixed, and fluorescent antibodies added. Detection was done with CTL equipment, and antibody titers were calculated using the Reed-Muench formula, with ≥30 considered positive.

### 2.10. FAMA

Antigen slides were prepared using varicella-zoster virus (VZV)-infected 2BS cells (Changchun Qijian Biological Products Co., Ltd., Changchun, China). Serum samples diluted in PBS (20 µL per sample) were applied to the antigen-coated slides and incubated in a humidified chamber at 37 °C for 1 h. After three washes with PBS (5 min per wash), slides were air-dried at room temperature. A light-protected working solution containing 0.01% Evans Blue and FITC-conjugated goat anti-mouse IgG (1:200 dilution, 10 µL per well) was added to the slides. Following a second incubation in the dark at 37 °C for 1 h, slides were washed and dried as described above. Samples were examined under an inverted fluorescence microscope for final evaluation. A positive result was defined by the presence of continuous circumferential fluorescence on the cell membrane, whereas the absence of fluorescent rings or only background signals without specific staining was classified as negative.

### 2.11. Hematoxylin and Eosin (H&E) Staining

The HE staining experiment was conducted by Wuhan Service Biotechnology Co., Ltd. (Wuhan, China), encompassing tissue sampling, fixation, embedding, and the preparation of both paraffin and frozen sections. The paraffin sections were sequentially immersed in a deparaffinization agent, anhydrous ethanol, and 75% ethanol, followed by rinsing with distilled water. The frozen sections were allowed to reach room temperature before being fixed with the appropriate tissue fixative. Subsequently, the sections were treated with HD constant staining pre-treatment solution and stained with hematoxylin, followed by rinsing and treatment with differentiation and bluing solutions. Finally, the sections underwent eosin staining, dehydration, and xylene treatment, and were sealed with a neutral mounting medium, completing the microscopy examination and image acquisition analysis.

### 2.12. Statistical Analysis

Statistical analysis of the ELISA experiment results was performed using GraphPad Prism 10.2. We used one-way ANOVA to assess differences between experimental groups and applied Tukey’s multiple comparisons test to compare group means. Flow cytometry results were expressed as the percentage of positive cells, with statistical analysis also conducted using GraphPad Prism 10.2. *p*-values for each group were reported as follows: * *p* < 0.05; ** *p* < 0.01, *** *p* < 0.001; ****, *p* < 0.0001; ns, not significant, *p* > 0.05.

## 3. Results

### 3.1. The Construction, Expression, and Purification of Protein Nanoparticles

**Figure 1:**
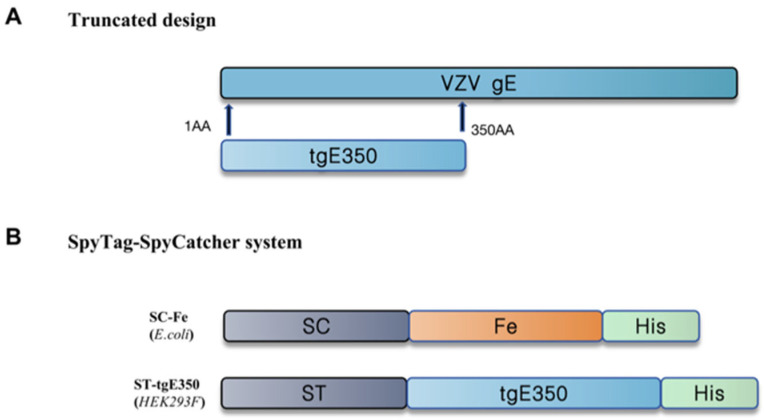
Schematic diagram of the construction of recombinant plasmids. (**A**) The gE protein truncation design of VZV was taken from gE gene 1-350AA; (**B**) tgE350 and Fe were designed using SpyTag-SpyCatcher covalent coupling. SC stands for SpyCatcher and ST stands for SpyTag.

Human ferritin (Fe) was selected as the nanocarrier platform for HZ vaccine development. Ferritin from bacteria (Pyrococcus furiosus and Helicobacter pylori ferritins), which poses a risk for in vivo application, elicits a stronger immune response, is damaging to experimental animals, and is susceptible to false positives in immunoassays, compared to human ferritin. The SpyTag/SpyCatcher system, derived from Streptococcus pyogenes, was employed to covalently link Fe and tgE350. The schematic diagram of the plasmid construction is shown in Figure 1, and the recombinant plasmids pET28a-SC-Fe-BL21 and pcDNA3.4-ST-tgE350-His were successfully constructed through validation.

**Figure 2:**
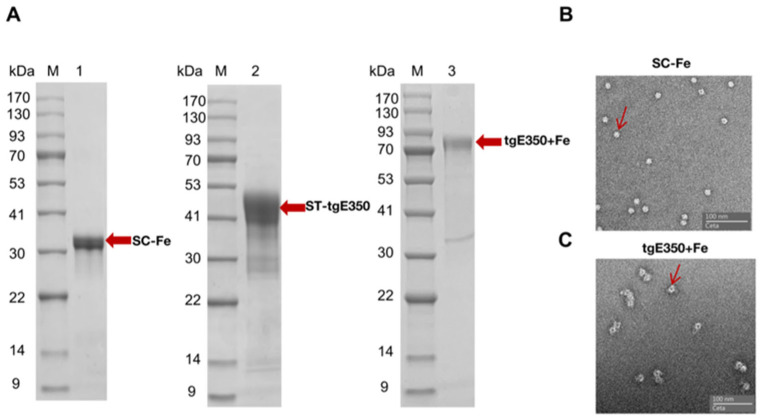
Validation of tgE350 + Fe nanoparticle protein. (**A**) M represents Protein Marker, 1: SDS-PAGE analysis of SC-Fe, 2: SDS-PAGE analysis of ST-tgE350, 3: SDS-PAGE analysis of tgE350 + Fe; (**B**) Negative-staining EM of SC-Fe. (**C**) Negative-staining EMs of tgE350 + Fe. The red arrows on the bands indicate the positions of the respective target proteins.

The two plasmids were expressed using *E. coli* BL21 and HEK293F cells, respectively. Optimal soluble expression of SC-Fe protein was observed in the culture supernatant with minimal contaminating proteins when induced with 1 mM IPTG at 16 °C. The purification process of the recombinant proteins was then employed for the isolation and purification of the recombinant proteins by means of nickel column chromatography, utilizing the properties of the His tag. The proteins were eluted by different concentrations of imidazole, and the correctness and purity of the eluted proteins were identified by SDS-PAGE. The purification of recombinant proteins showed that the target proteins SC-Fe and ST-tgE350 were eluted at 300 mM imidazole concentration with more than 95% purity. As shown in Figure 2A, distinct bands appeared at 34 kDa and 42 kDa, consistent with the predicted protein sizes. The purified SC-Fe was then mixed with ST-tgE350 in a 1:1 ratio without any enzyme. The formation of an intermolecular isopeptide bond between SC and ST results in irreversible coupling of ferritin to the antigen tgE350. Finally, tgE350 + Fe nanoparticles were collected by further purification using size exclusion chromatography (SEC) and then concentrated. The tgE350 + Fe protein concentration was determined to be approximately 1.52 mg/mL by BCA, and the nanoparticle size and morphology were then verified using SDS-PAGE and negative staining transmission electron microscopy. The SDS-PAGE electropherogram exhibited single and clear bands, with migration positions corresponding to the anticipated molecular weight of 76 kDa (Figure 2A). These results indicate that the nanoparticles possessed high purity with monodisperse molecular weight characteristics. Transmission electron microscopy (TEM) analysis further revealed uniformly distributed spherical nanostructures, displaying diameter measurements predominantly below 20 nm (Figure 2B,C).

### 3.2. tgE350 + Fe Nanoparticle Vaccine Elicits Robust Humoral Immune Responses in BALB/c Mice

**Figure 3:**
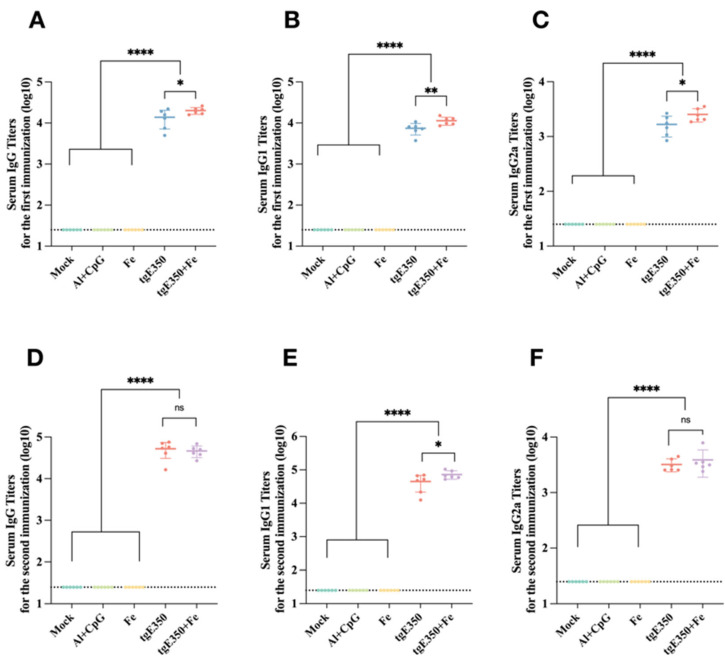
Humoral immune response in vaccinated BALB/c mice. (**A**–**C**) ELISA was employed to measure the gE-specific IgG, IgG1, and IgG2a titers in serum collected two weeks after the first immunization, induced by different immunological strategies. The titers shown in (**A**–**C**) are presented in log10 format. (**D**–**F**) ELISA was employed to measure the gE-specific IgG, IgG1, and IgG2a titers in serum collected two weeks after the second immunization, induced by different immunological strategies. The titers shown in (**D**–**F**) are presented in log10 format. *, *p* < 0.05; **, *p* < 0.01; ****, *p* < 0.0001, and ns, *p* > 0.5.

Serum levels of IgG and its subclasses in mice immunized with tgE350 + Fe and tgE350 nanoparticles were measured by ELISA. Fe, PBS, and Al/CpG were included as three control groups to evaluate humoral immune responses induced by the two nanoparticle formulations. Results showed that all experimental groups produced antigen-specific IgG antibodies in serum collected 14 days after the primary immunization. Among them, the nanoparticle protein groups elicited the highest IgG antibody titers, with statistically significant differences compared to all three control groups (*p* < 0.0001) (Figure 3A). Furthermore, after the first immunization, the tgE350 + Fe nanoparticle vaccine induced significantly higher levels of serum-specific IgG1 and IgG2a antibodies than the control groups (*p* < 0.05). This suggests the nanoparticle vaccine can trigger broad immune responses rapidly post-initial immunization, offering faster and stronger protection.

Serum antigen-specific antibody titers were quantified by ELISA two weeks after booster immunization. Following the second immunization, mice vaccinated with tgE350 + Fe exhibited significantly elevated titers of all three antibodies compared to the control group (*p* < 0.0001). The tgE350 + Fe group also showed a significantly higher IgG1 titer than the tgE350 group (*p* < 0.05) (Figure 3E), while no significant differences were observed in total IgG or IgG2a titers between the two nanoparticle groups (Figure 3D,F). Although the IgG2a level did not reach statistical significance, its mean value in the tgE350 + Fe group (3882) exceeded that of the tgE350 group (3207). The observed difference from the first immunization could be attributed to limited sample size leading to statistical variation, or a potential catch-up IgG2a response elicited in the tgE350 group following the booster dose.

**Figure 4:**
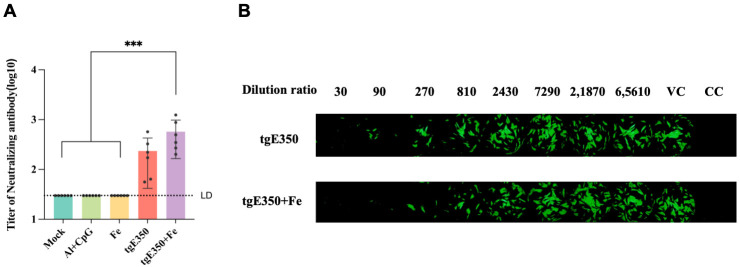
Microneutralization Antibody Titers. (**A**) The microneutralization assay measured the neutralizing antibody titers for each group. (**B**) Illustrative images of neutralization titers are shown, with “VC” representing virus control and “CC” representing cell control. One-way analysis of variance (ANOVA) was used to examine the data, and Tukey’s multiple comparisons test was used to compare each group’s mean with the means of all other groups. ***, *p* < 0.001.

The microneutralization assay, which measures the titer of neutralizing antibodies in serum, directly evaluates the virus-neutralizing ability of vaccine-induced immune responses and is a key indicator for assessing vaccine protective effects. In this study, the protective efficacy of nanoparticle proteins was further verified by microneutralization antibody assays. During the experiment, the focus was on evaluating the impact of nanoparticle vaccine treatment on the titer of neutralizing antibodies in mouse serum. As shown in Figure 4A, the experimental results indicated that in the serum after the second immunization, the tgE350 + Fe group had the highest titer of neutralizing antibodies, with a mean of approximately 575. This was higher than that in the tgE350 group and showed a statistically significant difference compared to the three control groups (*p* < 0.001).

**Figure 5:**
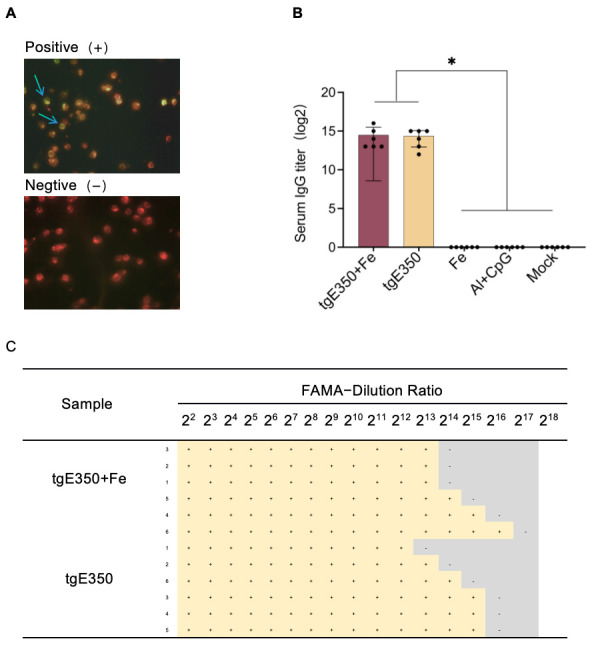
FAMA (Fluorescent Antibody to Membrane Antigen) Results. (**A**) Representative Fluorescent Images from FAMA Assay: positive control (Left picture) and negative control (right picture) (**B**) Serum IgG antibody levels in immunized mice; (**C**) Quantitative analysis of FAMA assay results from immunized mouse sera. Positive results (+) and negative results (−) indicate specific fluorescence at each serum dilution. One-way analysis of variance (ANOVA) was used to examine the data, and Tukey’s multiple comparisons test was used to compare each group’s mean with the means of all other groups. *, *p* < 0.05.

The Fluorescent Antibody to Membrane Antigen (FAMA) assay, a classic method for detecting VZV-specific antibodies, is widely used in virology. In this study, the FAMA assay, recognized as the gold standard for detecting specific IgG antibodies, was employed to measure IgG antibody levels in the tgE350 + Fe and tgE350 groups. This technique interprets results based on the continuous fluorescent signals formed by antigen–antibody complexes on the cell membrane surface. Positive samples (infected cell line 2BS) display complete peripheral fluorescent halos, while negative controls show no fluorescence (Figure 5A). The FAMA quantitative analysis was performed by serial dilution (dilution rate of 2^2^–2^18^) of mouse serum after immunization. The highest dilution at which cells display complete green fluorescent halos was defined as the endpoint titer. As shown in Figure 5B, both the tgE350 + Fe and tgE350 groups exhibited similar IgG antibody titers, which were significantly higher than those in the control group (*p* < 0.5).

### 3.3. tgE350 + Fe Nanoparticle Vaccine Drives Cell-Mediated Immunity in BALB/c Mice

**Figure 6:**
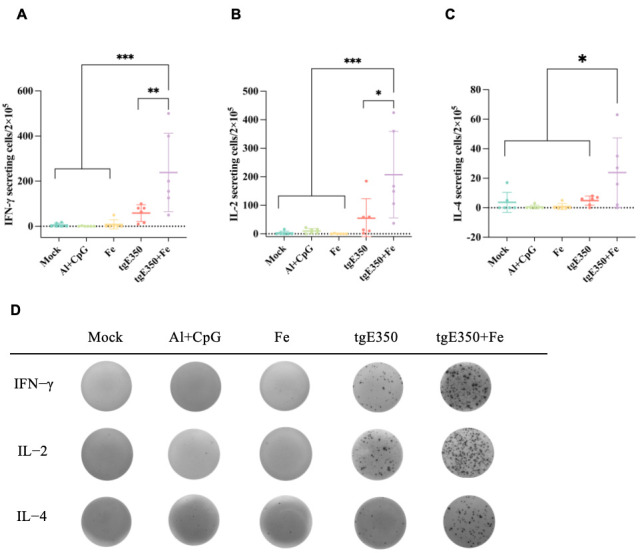
Levels of Cytokine-Secreting Lymphocytes in Mice Immunized with Nanoparticle Vaccines (**A**–**C**) ELISpot was used to identify the IFN-γ, IL-2 and IL-4 that splenocytes released after being stimulated with 10 μg/mL gE. N = 6 (**A**–**C**), where a single mouse is represented by each dot. (**D**) Representative pictures of splenocytes that produced IFN-γ, IL-2 and IL-4 using ELISpot. One-way analysis of variance (ANOVA) was used to examine the data, and Tukey’s multiple comparisons test was used to compare each group’s mean with the means of all other groups. *, *p* < 0.05; **, *p* < 0.01; and ***, *p* < 0.001.

**Figure 7:**
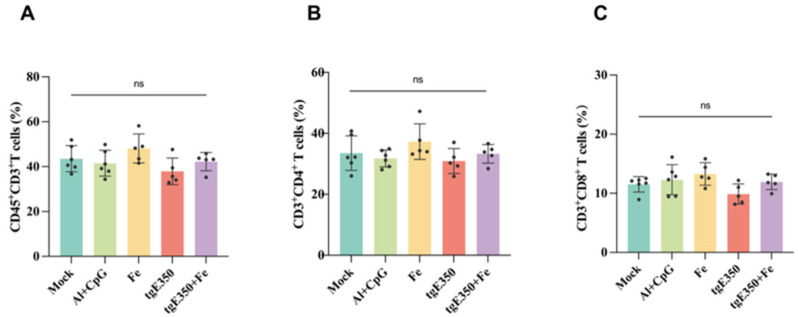
FCM Analysis of Different T Cells proportion in tgE350 + Fe Nanoparticle Vaccinated BALB/c Mice. (**A**) The percentage of CD3^+^T lymphocytes within the total leukocyte population in splenocytes. (**B**) The percentage of CD4^+^T cells in splenocytes. (**C**) The percentage of CD8^+^T cells in splenocytes. ns, *p* > 0.5.

The gE protein serves as the primary target for VZV-specific CD4^+^ T cell responses. To evaluate whether nanoparticle vaccines could also elicit robust T cell responses, we employed ELISpot and flow cytometry (FCM) to analyze the distribution of T cell subsets and their cytokine secretion profiles in the spleens of mice vaccinated (Figure 6 and Figure 7). Spleen cells were collected from mice two weeks following the second immunization and analyzed for cytokine production to quantify the magnitude of vaccine-induced cell-mediated immune responses. Our analysis revealed that the tgE350 + Fe group exhibited significantly higher IFN-γ-secreting cells (239/2 × 10^5^ lymphocytes) compared to the tgE350 group (58/2 × 10^5^ lymphocytes), with a *p* < 0.01 indicating statistical significance. Similarly, for IL-2 secretion, the tgE350 + Fe group demonstrated substantially more IL-2-secreting cells (207/2 × 10^5^ lymphocytes) than the tgE350 group (55/2 × 10^5^ lymphocytes). The differences between the tgE350 + Fe group and the three control groups were highly significant (*p* < 0.001). We also assessed IL-4 secretion in spleen cells using ELISpot assays. The tgE350 + Fe group showed fewer IL-4-secreting cells (24/2 × 10^5^ lymphocytes), but still exhibited statistically significant differences compared to other groups (*p* < 0.05). To analyze T cell phenotypes and cytokine secretion levels, we collected mouse splenocytes 14 days after the second immunization and performed flow cytometry analysis. The tgE350 and tgE350 + Fe groups generated similar proportions of CD3^+^ T, CD4^+^ T, and CD8^+^ T cells (Figure 7A–C).

### 3.4. Safety of tgE350 + Fe Nanoparticle Vaccine in Mice

**Figure 8:**
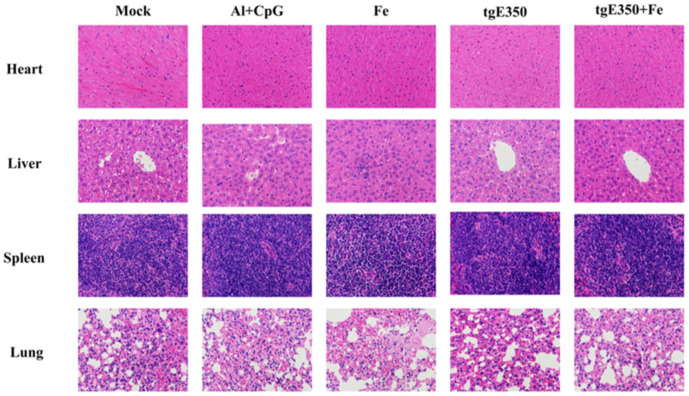
Safety of Nanoparticles Vaccinated BALB/c Mice. HE staining was used to observe the pathological sections of heart, liver, spleen and lung in different groups of mice.

The HE staining was performed on the pathological sections of the heart, liver, spleen and lung of mice to observe whether the recombinant immunogen used in the experiment was safe for mice. Compared with the tissue sections of mice without immune recombinant protein, no obvious lesions were observed in the tissue sections of major organs of mice immunized with Fe, tgE350 and tgE350 + Fe (Figure 8). The results demonstrated that Fe protein and nanoparticle immunogen are safe in mice.

## 4. Discussion

Varicella-zoster virus (VZV) affects about one-third of the population [19]. After the initial infection, it remains latent and may reactivate as herpes zoster (HZ) in older people or those with weakened immune systems [20,21]. The incidence of herpes zoster rises with age. Varicella zoster virus is highly contagious and frequently causes outbreaks of chickenpox, although vaccination has significantly reduced cases [22]. There are two shingles vaccine options: the live attenuated Zostavax vaccine and the more effective recombinant Shingrix. Shingrix is more than 90% effective in preventing HZ in adults over the age of 50 years, including immunocompromised individuals. Common side effects of Shingrix include pain at the injection site, fatigue, and mild fever [23]. Overall, the widespread use of the shingles vaccine has significantly reduced the incidence of herpes zoster and associated complications. This is critical for improving public health outcomes [14].

As is well-known, gE, a major VZV virulence factor, is essential for viral replication in the skin and cell-to-cell spread. It interacts with other proteins [24], promoting secondary envelopment of viral particles for cell-to-cell transmission and boosting viral neurovirulence [19]. Being the most abundant glycoprotein on the viral particle envelope and infected cell surface, gE is a protective antigen inducing both cellular and humoral immunity post-vaccination. It is also the main target for VZV-specific CD4 ^+^ T cell responses [21]. Recent studies show truncated vaccines can precisely stimulate the desired immune response, aiding strong immunological memory against specific pathogens. Moreover, truncated vaccines usually have fewer side effects, enhancing vaccine safety and making them a key focus in modern vaccine development [25]. Our team found that truncated gE (tgE350) induces higher neutralizing antibodies than gE, so tgE350 was chosen as the target for our vaccine design (Truncated VZV gE Induces High-Titer Neutralizing Antibodies in Mice).

This study successfully developed a VZV truncated gE antigen (tgE350)-based nanoparticle vaccine (tgE350 + Fe). Through the SpyTag/SpyCatcher system, the antigen was covalently anchored on human ferritin nanoparticles. Results showed that this vaccine significantly enhanced VZV-specific neutralizing antibodies and cellular immune responses in mice, while not causing tissue damage. It demonstrates good safety and immunogenicity. The discussion covers vaccine design, immunological advantages, safety, and future research directions.

The experiment used human ferritin as a nanoparticle carrier. Compared with bacterial ferritin, it has higher biocompatibility, avoiding non-specific immune responses or detection interference from heterologous proteins [26]. Antigen covalent linking via the SpyTag/SpyCatcher system ensures nanoparticle stability and antigen display density and prevents antigen conformational changes caused by traditional chemical conjugation [27]. Electron microscopy confirmed that tgE350 + Fe nanoparticles are about 20 nm in diameter, a size conducive to transport via the lymphatic system and efficient uptake by antigen-presenting cells (APCs).

In humoral immunity, the nanoparticle vaccine tgE350 + Fe demonstrated significant advantages, which indicate the nanoparticle vaccine shows significant advantages in enhancing the speed and intensity of immune responses, providing effective protection in a shorter time. IgG1 antibody production is typically associated with Th2 immune responses, which mainly reflect humoral immunity and play a key role in neutralizing extracellular pathogens and initial infections [28,29]. This implies nanoparticle display significantly enhances B-cell receptor cross-linking and activation through multivalent antigen epitope presentation.

High-level neutralizing antibodies are typically seen as a key sign of vaccine effectiveness. Detecting increased neutralizing antibodies after vaccination is crucial for confirming sufficient immune protection. Further neutralizing antibody (NAb) tests showed that after the second immunization, the nanoparticle group’s NAb titer was 1.7 times higher than that of the tgE350 group, showing its potential as an effective vaccine candidate. This aligns with prior research on ferritin nanoparticles in flu vaccines. Kanekiyo et al. fused influenza virus HA with ferritin genes to create HA-ferritin nanoparticles. Immunization with this flu nanoparticle vaccine induced hemagglutination inhibition antibody titers about 11 times higher than those from inactivated vaccines. Moreover, it triggered neutralizing antibody responses to two highly conserved domains, enhancing the efficacy and breadth of influenza virus immunity [18].

In terms of cellular immunity, the tgE350 + Fe group showed a significant increase in the number of lymphocytes secreting IFN-γ and IL-2 (*p* < 0.0001), indicating effective activation of Th1-type cellular immune responses. This aligns with the characteristics of the gE protein as the major CD4^+^ T-cell epitope of VZV and is consistent with the mechanism of the Shingrix vaccine, which relies on CD4⁺ T-cell help. The lower secretion of IL-4 and the Th1-biased immune response are more advantageous for combating intracellular viral infections. IFN-γ, a hallmark cytokine of Th1 cells, plays a key role in cellular immunity, while IL-2 is essential for T-cell proliferation, survival, and differentiation. Their synergistic action drives the Th1 immune response, which can activate broader and longer-lasting immune protection [30,31]. Compared to tgE350, the nanoparticle vaccine more effectively enhances the Th1 response dominated by IFN-γ and IL-2, facilitating the formation of long-term cellular immune memory. For elderly individuals, boosting the Th1 response is key to preventing the reactivation of latent viruses. Studies have confirmed that a robust Th1 response is crucial for the successful prevention of herpes zoster. The nanoparticle vaccine may enhance T-cell activation by more effectively presenting multiple immunogenic epitopes due to its structural properties, thereby promoting stronger and more specific helper T-cell responses and strengthening cellular immunity. The results of this study showed no significant differences in the distribution and function of multiple T-cell subsets among the treatment groups, suggesting that vaccine treatment had limited effects on these specific T-cell subsets. It should be noted that our experimental design has certain limitations: while it aimed to assess natural immune responses in vivo, the lack of in vitro stimulation may have affected the comprehensive evaluation of T-cell responses, potentially restricting a full understanding of vaccine-induced immune responses.

Research on Fe protein nanoparticle vaccines is gaining attention [32]. This increased interest is closely related to the mechanisms of ferritin nanoparticles, which promote APC uptake via size effects [18], activate TLR pathways to boost Th1 responses [33], and prolong the intensity and duration of immune reactions in the body. These nanoparticles extend antigen activity, enhance stability, and allow the immune system sufficient time to generate effective responses [34].

Although the currently marketed Shingrix vaccine demonstrates high protective efficacy, its widespread adoption is limited by local reactions associated with the AS01_B_ adjuvant (including injection site pain with an incidence exceeding 80%) and relatively high production costs [35]. This study used an Al/CpG adjuvant system that combines the antigen-releasing properties of aluminum adjuvants with the Th1 immune-activating effects of CpG. It achieved strong immune responses while ensuring safety. Histopathological examination detected no signs of inflammation or damage in the major organs of mice immunized with tgE350 + Fe. Moreover, statistical comparison showed no significant difference from the control group. Collectively, these results confirm the biosafety of the vaccine components. In addition, SC-Fe and ST-tgE350 were produced using a dual expression system in *E. coli* and mammalian cells, respectively, and self-assembled into nanoparticles by covalent coupling without the need for exogenous enzymes or specialized buffers. This significantly reduces production costs and lays the foundation for large-scale production. This production strategy establishes a novel approach for HZ vaccine development through nanoparticle-mediated antigen presentation, while simultaneously providing a critical reference for the design of self-assembling nanoparticle vaccine platforms [36].

Although the nanoparticle vaccine (tgE350 + Fe) constructed in this study induced antibody titers comparable to those of the soluble antigen (tgE350) under standard immunization regimens, it elicited a stronger Th1-biased cellular immune response. This qualitative shift in immune response may be particularly important for controlling viral latency and reactivation. It should be noted that the conclusions of this study have two main limitations. First, under the current experimental conditions, the advantage of nanoparticles in enhancing peak antibody levels has not yet been demonstrated. Second, the study did not include animal challenge experiments to directly confirm its protective efficacy. The former suggests that future work should focus on optimizing antigen dose and adjuvant strategies to further explore the platform’s potential. The latter is due to the current lack of an ideal small animal infection model for VZV. However, the high levels of neutralizing antibodies and the balanced Th1/Th2 immune response confirmed in this work are both recognized as key correlates of protection. Therefore, the results of this study lay a solid immunological foundation for the further development of this vaccine candidate. Future efforts should focus on establishing or employing more suitable challenge models to directly evaluate its protective effect and exploring simplified formulations with lower doses or without additional adjuvants, thereby highlighting its practical advantages as a next-generation vaccine platform.

## 5. Conclusions

In this study, the HZ nanoparticle vaccine (tgE350 + Fe) was successfully constructed based on the SpyTag/SpyCatcher covalent conjugation system, and its immunogenicity and safety were evaluated in a BALB/c mouse model. The results showed that the nanoparticle vaccines were all successfully expressed and formed stable nanostructures, and the tgE350 + Fe group showed the best response in humoral and cellular immune responses. The tgE350 + Fe-immunized group induced the highest number of IFN-γ- and IL-2-secreting lymphocytes and produced the highest titer of neutralizing antibodies, which was significantly better than the tgE350 group. None of the vaccine groups induced significant organ pathological damage or significant lymphocyte population changes, indicating a favorable safety profile. This study demonstrated that the vaccine designed based on SpyTag/SpyCatcher-designed Fe protein nanoparticles could effectively activate both humoral and cellular immune responses, which provides an important experimental basis for the optimal design of the next-generation herpes zoster vaccine.

## Data Availability

All the data from the study are available from the corresponding author upon reasonable request.
